# Optimization of graphene polypyrrole for enhanced adsorption of moxifloxacin antibiotic: an experimental design approach and isotherm investigation

**DOI:** 10.1186/s13065-024-01208-0

**Published:** 2024-06-13

**Authors:** Sara Ishaq, Ahmed H. Nadim, Sawsan M. Amer, Heba T. Elbalkiny

**Affiliations:** 1 Analytical Chemistry Department, Faculty of Pharmacy, MSA University: October University for Modern Sciences and Arts, Cairo, Egypt; 2 Pharmaceutical Analytical Chemistry Department, Faculty of Pharmacy, Cairo University, Cairo, Egypt

**Keywords:** Adsorption, Wastewater treatment, Nanoparticles, Graphene polypyrrole, Moxifloxacin HCl

## Abstract

The presence of antibiotics in water systems had raised a concern about their potential harm to the aquatic environment and human health as well as the possible development of antibiotic resistance. Herein, this study investigates the power of adsorption using graphene-polypyrrole (GRP-PPY) nanoparticles as a promising approach for the removal of Moxifloxacin HCl (MXF) as a model antibiotic drug. GRP-PPY nanoparticles synthesis was performed with a simple and profitable method, leading to the formation of high surface area particles with excellent adsorption properties. Characterization was assessed with various techniques, including Fourier-transform infrared spectroscopy (FTIR), scanning electron microscopy (SEM), X-ray diffraction (XRD) and Brunauer–Emmett–Teller (BET). Box-Behnken experimental design was developed to optimize the adsorption process. Critical parameters such as initial antibiotic concentration, nanoparticle concentration, and pH were investigated. The Freundlich isotherm model provided a good fit to the experimental data, indicating multilayer adsorption of MXF onto the GRP-PPY-NP. As a result, a high adsorption capacity of MXF (92%) was obtained in an optimum condition of preparing 30 μg/mL of the drug to be adsorbed by 1 mg/mL of GRP-PPY-NP in pH 9 within 1 h in a room temperature. Moreover, the regeneration and reusability of GRP-PPY-NP were investigated. They could be effectively regenerated for 3 cycles using appropriate desorption agents without significant loss in adsorption capacity. Overall, this study highlights the power of GRP-PPY-NP as a highly efficient adsorbent for the removal of MXF from wastewater as it is the first time to use this NP for a pharmaceutical product which shows the study's novelty, and the findings provide valuable insights into the development of sustainable and effective wastewater treatment technologies for combating antibiotic contamination in aquatic environments.

**Supplementary Information:**

The online version contains supplementary material available at 10.1186/s13065-024-01208-0.

## Introduction

Waterborne disease development, poor sanitation and low quality of water supply are all posing global challenges because of the high demand by the huge rising populations [21]. The expectations refer that the average water demand per person will drop by a third, which may lead to a health disaster if inadequate measures and steps are not taken seriously to avoid this situation. Pharmaceuticals are an important group of potential micropollutants that have recently attracted much attention and have been found in trace amounts in a variety of environmental water samples, including surface water, ocean, groundwater, and effluents from sewage treatment plants [31, 32, 44],and [15]. Unfortunately, the existing water treatment technology is still considered inadequate for micropollutants elimination and removal, as they are not created to handle this specific class of pollutants [21]. Examples of such pharmaceutical active compounds are antibiotics which have a high biological activity, are extremely beneficial to humans, and have been at the forefront of prescribed and over-the-counter medications. Although they are extremely beneficial for human health, their presence in the environment nonetheless raises great concerns. According to Adeyemi et al. [1], the emergence of antibiotic resistance is the most important concern. Some isolated bacteria that were discovered in the sewage reactors of some treatment plants already showed signs of antibiotic resistance to powerful antibiotics such as fluoroquinolones (FQ) [1]. Since the COVID pandemic, an increase in their consumption worldwide has risen especially for MXF (S1, Supplemental Material) [16]. FQ antibiotics such as MXF are only partially metabolized in the body and are partially excreted in their pharmaceutically active form (> 50%). Therefore, the detected amount of FQ antibiotic residues in common wastewater treatment plant effluent has been increased. These concentrations may induce quinolone-resistant infections and persistent harm in aquatic organisms by applying selective pressure to microbial populations [37]. The inability of conventional wastewater treatment plants to get rid of the waste clearly shows the urgent need for cutting-edge technologies that can effectively deal with these compounds. The new techniques should ideally be able to remove pollutants, have low energy requirements, be cost-effective, be environmentally friendly, and be able to inactivate resistant pathogens. Adsorption is a type of tertiary stage of wastewater removal. It occurs by the transfer of the pollutant phase into another phase, the most common is that this phase is solid. It is used to remove dissolved impurities from water [35]. The adsorption technique has many advantages to be mentioned as it is easy to operate and design [10]. Also, it works at mild operation conditions and a wide range of pH with high efficiency. It requires a low energy in comparison to other methods making it an environmentally friendly method almost without any toxic products [19]. A lot of materials can be used as adsorbent as well, and the high surface area of adsorbent materials allows a high utilization and selectivity to certain contaminants or molecules. Finally, it can be easily regenerated and reused as it has a superior performance and robustness for consecutive cycles making it a cost-effective and affordable method [19]. Graphene nanoparticle (GRP-NP) is an intelligent two-dimensional (2D) carbon nanomaterials with one atom thickness of its own interesting electrical, electronic, optical, and mechanical properties, hence, received considerable interest in numerous industrial applications [12]. It was reported as a potent adsorbent in many research, a potential adsorbent for many applications in wastewater treatment due to its rich surface functional groups and high surface area [43]. Polypyrrole (PPY) alone is a conducting polymer and is extensively used in various applications due to its good environmental and thermal stability, high electrical conductivity, simple methods for production, and excellent redox characteristics. [7]. These intriguing characteristics offer a great substrate for the adsorption of contaminants onto charge centers. To properly recover antibiotics removal from aqueous wastewater discharge, a multifunctional adsorbent such as GRP-PPY-NP was designed and manufactured. Doping GRP with PPY to synthesize (GRP-PPY-NP) made a synergistic effect produced by combining these two materials as nanoparticles leading to improved characteristics and a multitude of possible applications. The importance of GRP-PPY-NP is their high electrical conductivity compared to GRP or PPY alone. Also, their extremely large surface area. Incorporating graphene into polypyrrole nanoparticles increases the effective surface area of the composite material. This enlarged surface area enables better interaction with other substances, such as gases or molecules, and drug delivery systems. Another importance is they can be tailored to exhibit biocompatible properties, making them suitable for biomedical applications. The surface of GRP-PPY-NP can be functionalized with biomolecules, drugs, or targeting moieties, enabling targeted drug delivery, bioimaging, and biosensing. Finally, the unique properties of GRP-PPY-NP make them promising candidates for environmental remediation. Their high electrical conductivity and large surface area facilitate efficient pollutant adsorption and degradation. GRP-PPY-NP-based nanocomposites can be used for the removal of heavy metals, organic pollutants, and water purification, thereby addressing various environmental challenges. It was reported that graphene and polypyrrole were combined to be used for only dyes [5] and ion removal [7]. The application of GRP-PPY-NP for the removal of complex high molecular weight organic molecules such as MXF has not been investigated yet. In the present work, GRP-PPY-NP was synthesized via a simple and green approach at room temperature. Then, applied as an efficient adsorbent to remove a pharmaceutical drug (MXF) from wastewater samples for the first time, which is considered the novelty of the work. Removal of MXF from wastewater was previously reported using different adsorption methods. However, either a long process of preparation with high costs, time consumption, or high temperature such as non-eco-friendly methods were adopted such as biochars [3], metal–organic framework (MOF) [46], natural zeolite [33], in-tube solid-phase microextraction using stainless steel tube by a syringe loaded with Fe_3_O_4_ nanoparticles [2], and removal by long synthesis process of NP [6], which considered complicated processes. The removal of other drugs by using different nanoparticles by adsorption was also performed but they didn’t achieve the same results as the current work did. Adsorption was previously performed and reported in different techniques as reported in [13, 14, 25–30, 38–40],and [42]. The purpose of the current research is the study MXF adsorption and removal from water samples by GRP-PPY-NP for the first time for a pharmaceutical drug which is considered novel by an eco-friendly analysis. The experimental conditions were optimized using the Box-Behnken statistical design. Such an approach would provide a facile and economic alternative for wastewater treatment protocols.

## Experimental

### Materials and chemicals

Moxifloxacin HCl (≥ 98%) was provided by Eva Pharma, Egypt. Polyethylene Glycol (PEG), Methanol, Acetonitrile (≥ 99%), HCl, and NaOH (33%) were provided by El Nasr Pharmaceutical Chemicals Co., Egypt. Pyrrole and graphene were supplied by Sigma Aldrich. Ammonium persulfate (APS) (≥ 98%) was provided by Alpha Chemical Group, Egypt. Distilled water (DW) was used for the preparation and washing of samples. A 5 mg/mL stock solution was prepared by adding 0.05 g of MFX HCl diluted to 10 mL of DW water as a solvent in a volumetric flask. A working solution was prepared by transferring 2 mL of the previous stock solution and then diluting it to 100 mL using DW water to obtain 100 μg/mL working solution.

### Instruments

Scanning electron microscopy (SEM) was performed using a Quanta FEG250 microscope with an accelerating voltage of 20.00 kV. Shimadzu IR Affinity-1 was used for Fourier transform infrared spectrometry (FTIR) measurements. Brunauer–Emmett–Teller (BET) was conducted using Quantachrome TouchWin™ version 1.21. X-ray diffraction (XRD) was analyzed using Shimadzu XRD 6000 diffractometer with CuKa1 radiation (k = 1.54056A°) operating at the voltage and current of 40.0 (kV) and 30.0 (mA), respectively. HPLC Agilent 1200 series, with a photodiode array detector, was used. The experimental design was performed using Design-Expert®11 (Stat-Ease Inc., Statistics Made Easy, Minneapolis, USA).

### Synthesis of GRP-PPY-NP

#### Preparation of polypyrrole (PPY-NP)

Synthesis was performed according to the previously reported method with minor modifications by [7], Briefly, 2 g of APS was dissolved in a beaker containing 50 mL of DW. Then, 0.60 mL of freshly distilled pyrrole was added with magnetic stirring for 1 h. After the polymerization process was completed, the washing process was executed using methanol three times followed by DW and then an air-drying process at room temperature was applied.

#### Preparation of graphene-polypyrrole nanoparticles (GRP-PPY-NP)

First, GRP-PEG dispersion was prepared by dissolving 200 mg of PEG-400 in a glass beaker containing 20 mL DW then 60 mg of graphene was dispersed. Then, 15 mL of GRP-PEG dispersion solution was added to 0.2 g pyrrole in a glass beaker and magnetically stirred for 15 min. APS previous prepared solution (0.68 g dissolved in 15 mL of 1 M HCl) was added dropwise to the previous solution with magnetic stirring for 1 h. After the polymerization process, the prepared composite was filtered and washed with DW and methanol. Then, the obtained composite was stirred with 50 mL of 1 M NaOH followed by filtration, and washing using the same procedure and air-drying process at room temperature.

### Characterization analysis for GRP-PPY-NP

Particle size and shape of synthesized NPs as morphology information were measured using the Scanning electron microscopy (SEM). Fourier transform infrared spectrometer (FTIR) was used for structural composition. Samples were scanned from 4000–400 cm^−1^. Characteristics such as the specific surface area of the NPs and average NP pore diameter were determined according to the Brunauer–Emmett–Teller (BET). The crystallography of NP patterns detected by X-ray diffraction (XRD).

### Reversed‑phase liquid chromatography

**Fig. 1:**
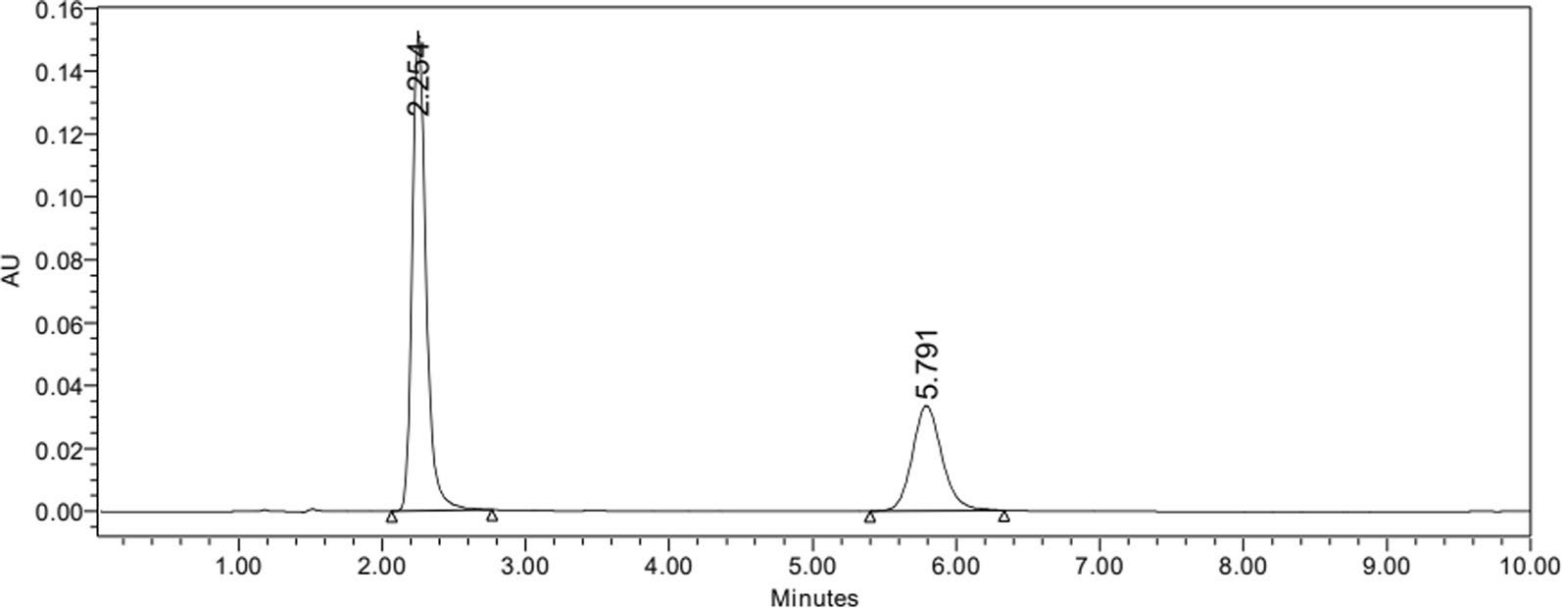
Chromatogram of Ciprofloxacin the internal standard and Moxifloxacin to obtain system suitability parameters

A previously reported method with minor modifications was used [34]. Kromasil C18 (150 × 4.6 mm) column was used. The optimum mobile phase is composed of 20 mM ammonium formate, pH adjusted to 4.0 with orthophosphoric acid and acetonitrile (70:30%v/v) at a flow rate of 1.3 mL/min, and detection at 295 nm. Samples were injected using a 20 µl fixed loop. Ciprofloxacin 50 μg/mL was the internal standard used. The calibration curve for MXF was constructed using a standard series covering a concentration range of 10–100 µg/mL. System suitability parameters were calculated according to US Pharmacopoeia [36], and interpreted from chromatogram peaks Fig. 1. Validation was carried out according to ICH Guidelines: Q2(R2) [18].

### Adsorption experiment

#### Screening of adsorption conditions

In 3 different beakers, 10 mL of working solution was withdrawn for each beaker. pH was adjusted to pH 5.0, 7.0, and 9.0 to test NP efficiency in both acidic, neutral, and alkaline medium. Then, different concentrations (0.5g/L, 1g/L, and 1.5g/L) of GRP-PPY-NP were added to each beaker. After 1 h, these samples were centrifuged and analyzed using an HPLC assay to check adsorption efficacy in the presence of many factors such as pH, the concentration of the NPs, and drug concentration. For optimization, ten beakers were prepared for further HPLC trials to optimize the best adsorption conditions.

#### Data analysis

Using a quadratic Box-Behnken design, the adsorption analysis was performed with the highest order polynomial and statistical analysis performed on the three responses (highest F-value and lowest P-value). A polynomial model was proposed as a function of independent variables and measured response choosing three factors: pH, adsorbent concentration, and drug concentration in three levels. For GRP-PPY-NP (adsorbent) concentration, the three levels were 0.5, 1, and 1.5 mg/mL. The second variable was MFX concentration with levels of 100, 30, and 65 μg/mL. Also, pH was chosen as 5.0, 7.0, and 9.0. Twelve runs were designed and performed based on operational variables.

#### Batch experiment

To investigate MXF adsorption on GRP-PPY-NP, batch experiments were conducted. For 1 h, 1 mg/mL of GRP-PPY-NP was prepared in pH 9 to adsorb 30 μg/mL which was the optimized condition for the best adsorption could the NP do.

### Adsorption isotherms

To give an insight into the adsorption process basic information on the interaction between the surface of the adsorbent and the adsorbate can be determined from several adsorption isotherms. Several isotherms are commonly used to characterize the drug performance and the best fit was obtained using the Freundlich model which assumes multilayer adsorption with heterogeneous surface energy systems and adsorbate–adsorbate interactions. The expression is as follows,

$${\text{Qe }} = {\text{ kfC}}^{{{{1}}/{{n}}}}$$

Where kf determines the Freundlich constant ([mg/g] [l/mg]1/n and n denotes the adsorption phenomena [41]. If n < 1, the system was chemisorption and n > 1, simulates the system to be physisorption [39, 40].

## Results and discussion

### Characterization analysis for GRP-PPY-NP

#### Fourier transforms infrared spectroscopy (FT-IR)

**Fig. 2:**
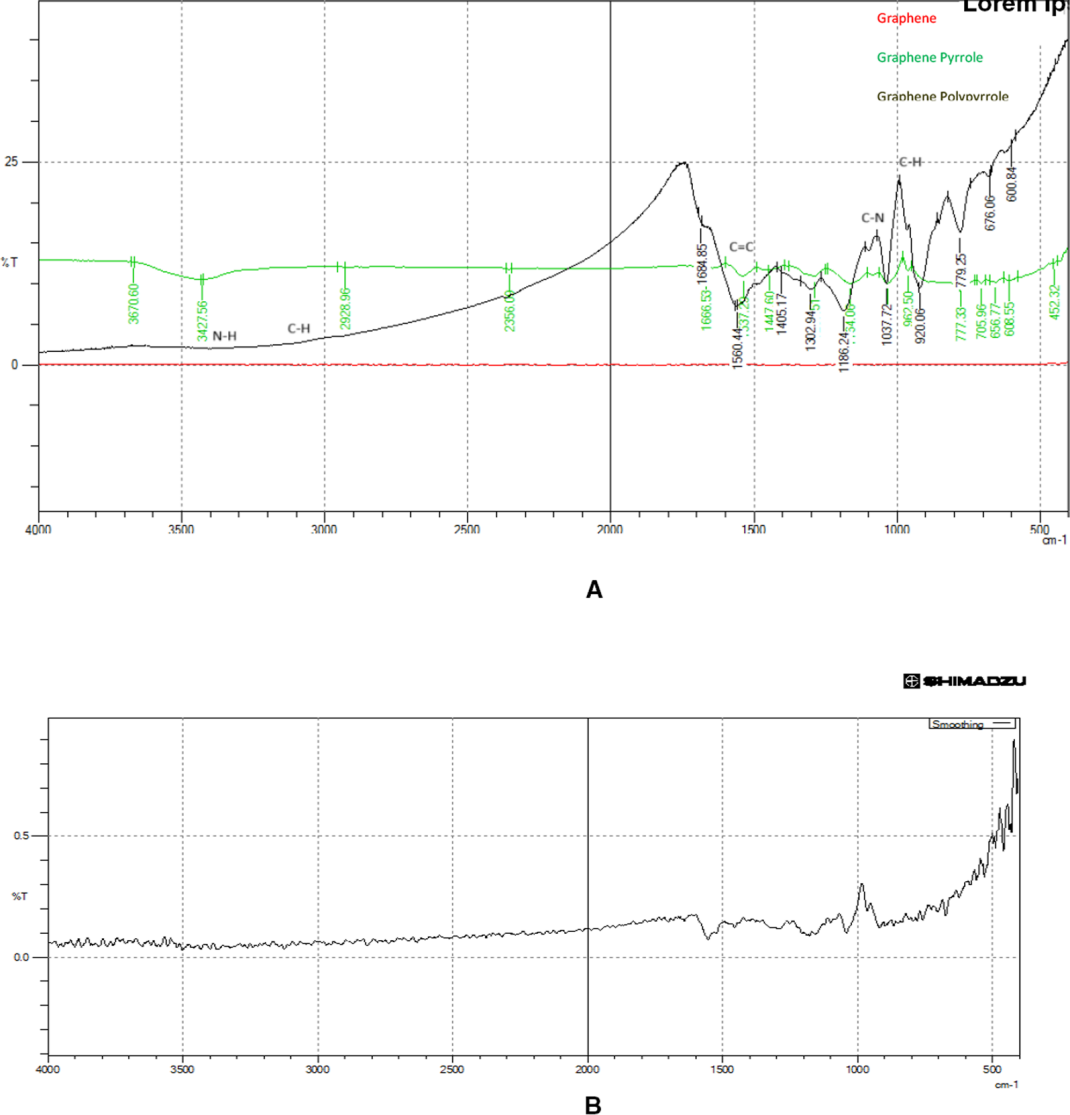
**a** IR scanning of graphene, graphene pyrrole, and graphene polypyrrole. **b** IR scanning of GRP-PPY-NP after MXF adsorption

Information on the chemical structure of nanoparticles and functional groups was obtained using FT-IR analysis. To detect if GRP-PPY-NP possesses all functional groups of GRP and PPY together, IR analysis was done for them all. As shown in Fig. 2a. The analysis ranged from 4000–400 cm^−1^ and the spectrum exhibited by the appearance of the C-H stretching vibration of the pyrrole ring appears at 3100–3000 cm^−1^. The N–H stretching vibration of the pyrrole ring appeared at around 3400–3300 cm^−1^. The C = C stretching vibration of the pyrrole ring appears around 1600–1500. The C-N stretching vibration of the pyrrole ring appears around 1300–1200 cm^−1^. The C-H bending vibration of the pyrrole ring appears at around 1000–900 cm^−1^ [6]. After MXF adsorption, the IR spectrum has changed adsorption causing shifts in characteristic peaks indicating interactions between MXF and the GRP-PPY-NP. For example, MXF forms hydrogen bonds and other chemical interactions with the functional groups present in the composite, which lead to shifts in the corresponding peaks. Some peaks associated with the NP material disappear or decrease in intensity due to the adsorption process. Also, the intensity of certain peaks in the FT-IR spectrum changed after MXF adsorption. This occurs due to the presence of MXF affecting the local environment of functional groups in the GRP-PPY-NP, leading to changes in their vibrational behaviour.(Fig. 2b).

#### Scanning *electron* microscope (SEM)

**Fig. 3:**
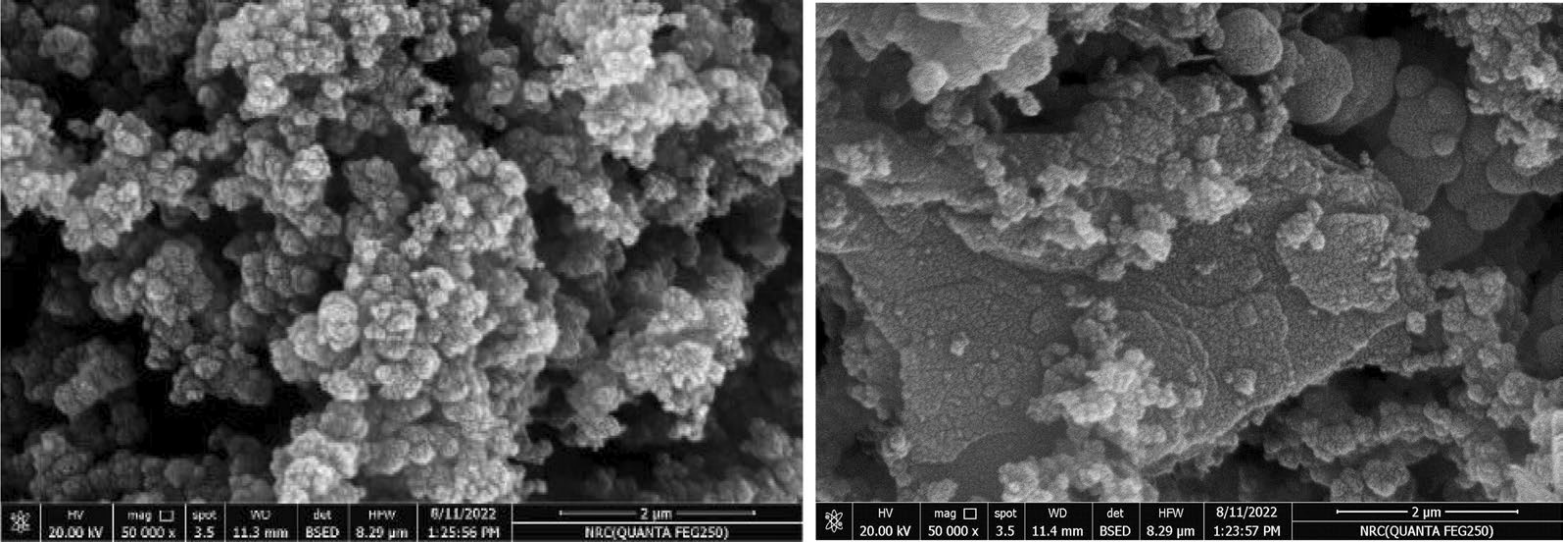
SEM of GRPPPY-NP

The morphological properties of GRP-PPY-NP adsorbent were studied using SEM. Nanoparticles' spherical and amorphous shape was detected to show a highly porous particle. (Fig. 3). Because of the huge number of interconnected pores, high porosity in GRP-PPY-NP shows some benefits such as large surface area compared to non-porous particles of the same size, so high quantities of adsorbate molecules would be captured and retained [11]. Other important advantages of high porosity are enhancing accessibility which allows the accessibility to the internal surface for more active adsorption, increasing the surface area, high selectivity, high versatility, and efficient mass transfer.

#### X-ray diffraction (XRD)

**Fig. 4:**
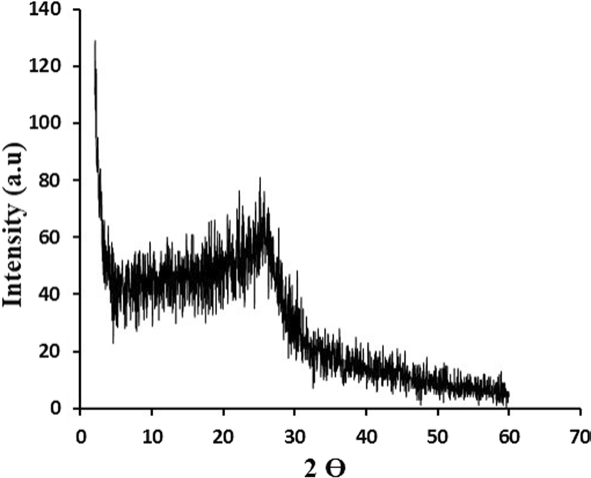
XRD of GRPPPY-NP

The structure of GRP-PPY-NP and its crystallinity were confirmed by exposing the samples to the XRD analysis, while the location of peaks will provide related information. As shown in Fig. 4, It was done by a scanning rate of 0.05°s–1 and 2θ in the range of 60°. The diffraction peaks can be well indexed to GRP-PPY-NP as a broad diffraction peak centered at 2θ = 25º exists in the XRD pattern indicating the presence of carbon in GRP [23]. A peak at 2θ = 20º indicates the presence of PPY [22]. A diffraction peak at 2θ = 20.8º indicates well blinding of GRP-PPY-NP and π–π stacking interactions between GRP and PPY.

#### Brunauer–Emmett–Teller analysis (BET)

**Fig. 5:**
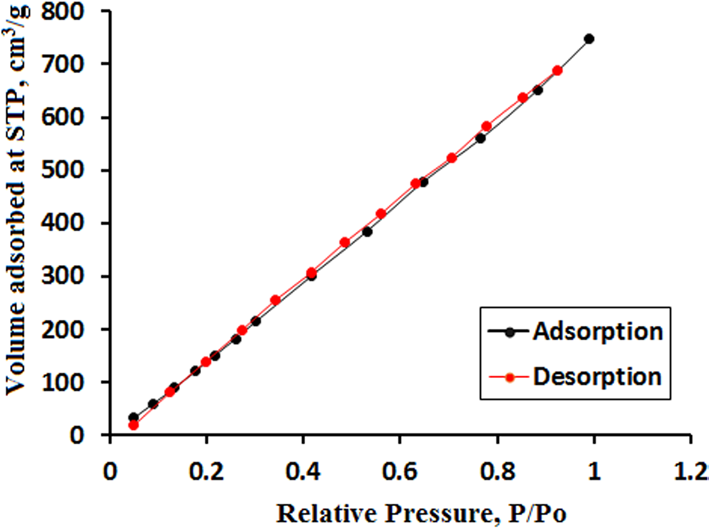
BET of GRPPPY-NP

BET analysis was used to estimate the specific surface area of NP while the pore size and pore volume of NP were estimated by the Barrett-Joyner-Halenda (BJH) model from the adsorption–desorption isotherm. Results showed that the surface area was 218.23 m^2^/g. The pore radius was 1.93 nm indicating a mesoporous nature of the composite with a total pore volume of 0.21 cc/g. Nitrogen adsorption–desorption isotherm showed type IV isotherm with H3 hysteresis loop which is characteristic of mesoporous materials (Fig. 5).

### Reversed-phase liquid chromatography

**Fig. 6:**
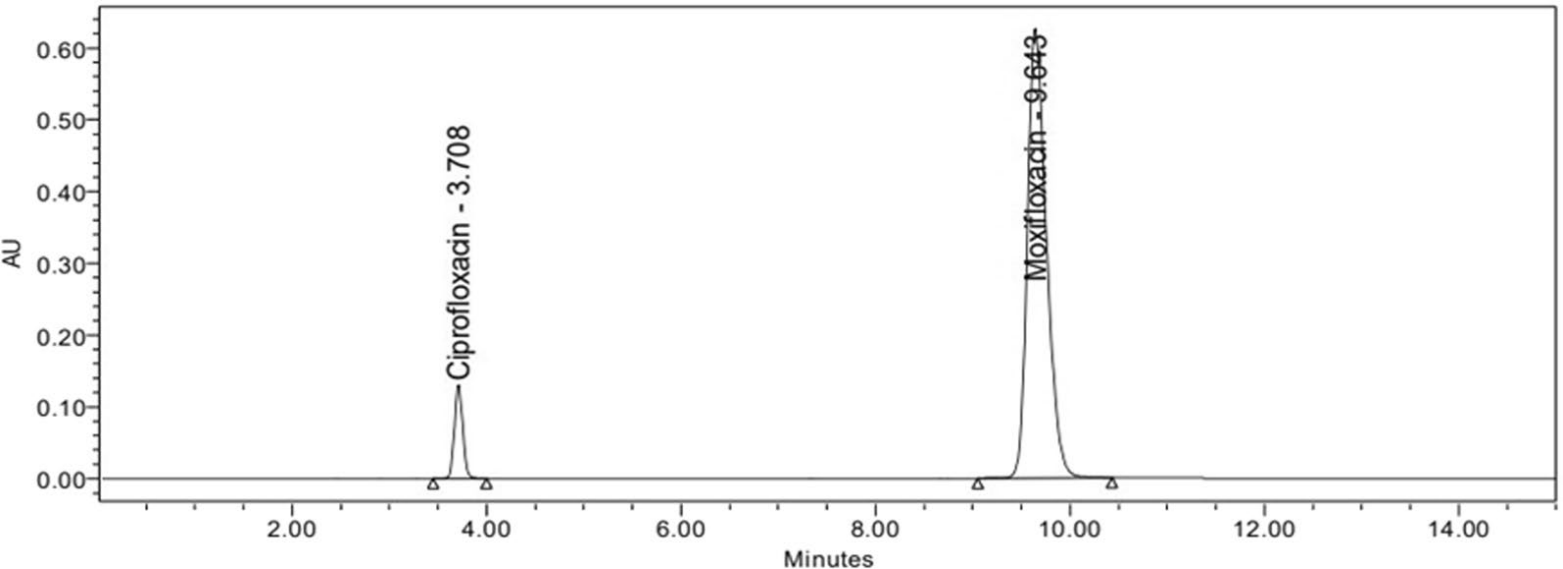
Chromatogram of MXF using Ciprofloaxcin as internal standard

According to the reported chromatographic method by [34], samples’ concentrations were determined using the proposed HPLC method. Before analysis, ciprofloxacin was used as an internal standard (Fig. 6) due to the similarity between its structure and MXF structure to decrease instrumental deviations. System suitability parameters were computed according to the US Pharmacopoeia (USP, 2021) (Table 1). Validation parameters (linearity, accuracy, precision, specificity, robustness, ruggedness) and regression equation were also summarized in (Table 2) according to ICH guidelines (ICH, 2022).

**Table 1:** Summary of system suitability for RP-HPLC assay:

System suitability parameters:	Ciprofloxacin	Moxifloxacin
Retention time (min)	2.254	5.791
USP Tailing	1.24	1.13
Resolution (Rs)	13.3	
Capacity factor (k')	0.502	2.86
Selectivity	5.69

**Table 2:** Summary of validation parameters for the reported RP-HPLC assay

Validation parameters	
Accuracy (mean ± SD)	99.97% ± 0.12
Precision (%RSD)	
Repeatability	0.86%
Intraday and interday assay	Intraday: 0.33% Interday: 0.60%
Linearity	
Regression equation	Y = 74.576x-17.466
Regression coefficient (R^2^)	0.999
Sensitivity	
LOD	0.35 µg/mL
LOQ	1.16 µg/mL
Ruggedness (%RSD)	0.52%
Robustness	
%RSD for different pH	0.48%
Flow rate	1.01%

### Experimental design

According to [9], the Box-Behnken statistical model (BBD) uses three levels per input variable and fits the output to a quadratic model with the highest order polynomial according to the experimental design results and the performed statistical analysis (Table 3). To evaluate the effect of each variable, a quadratic polynomial model was proposed as a function of independent variables (operational variables) and measured response (adsorption percentage). Three factors with three levels were chosen which were effectively responsible for changing of adsorption percentage. Factors were pH, adsorbent concentration, and drug concentration as mentioned before in a fixed time of 1 h and fixed room temperature to achieve an eco-friendly method. The model results obtained by experimental design are shown in the following equation:

**Table 3:** The factors and their levels used for Box-Behnken design

Run	A:pH Ca	B:Adsorbent conc mg/mL	C:drug conc μg/mL	Removal * % ± S.D
1	9	1	30	92.4 ± 1.34
2	7	1.5	100	64.65 ± 1.12
3	7	1	65	68.19 ± 1.26
4	7	1	65	67.99 ± 1.16
5	9	0.5	65	88.5 ± 1.28
6	5	0.5	65	50.98 ± 1.34
7	5	1	30	55.91 ± 1.22
8	5	1.5	65	51.99 ± 1.42
9	7	0.5	100	65.87 ± 1.26
10	7	1.5	30	70.87 ± 1.32
11	5	1	100	48.55 ± 1.22
12	9	1	100	90 ± 1.18

*Average of three readings

Removal percentage of MXF =  + 68.356, + 18.683 A, + 0.0362 B, − 2.2475C, − 0.8775 AB, + 1.24 AC, − 1.055 BC, + 2.7054 A^2^, − 1.6945 B^2^ and + 0.6529 C^2^.

**Fig. 7:**
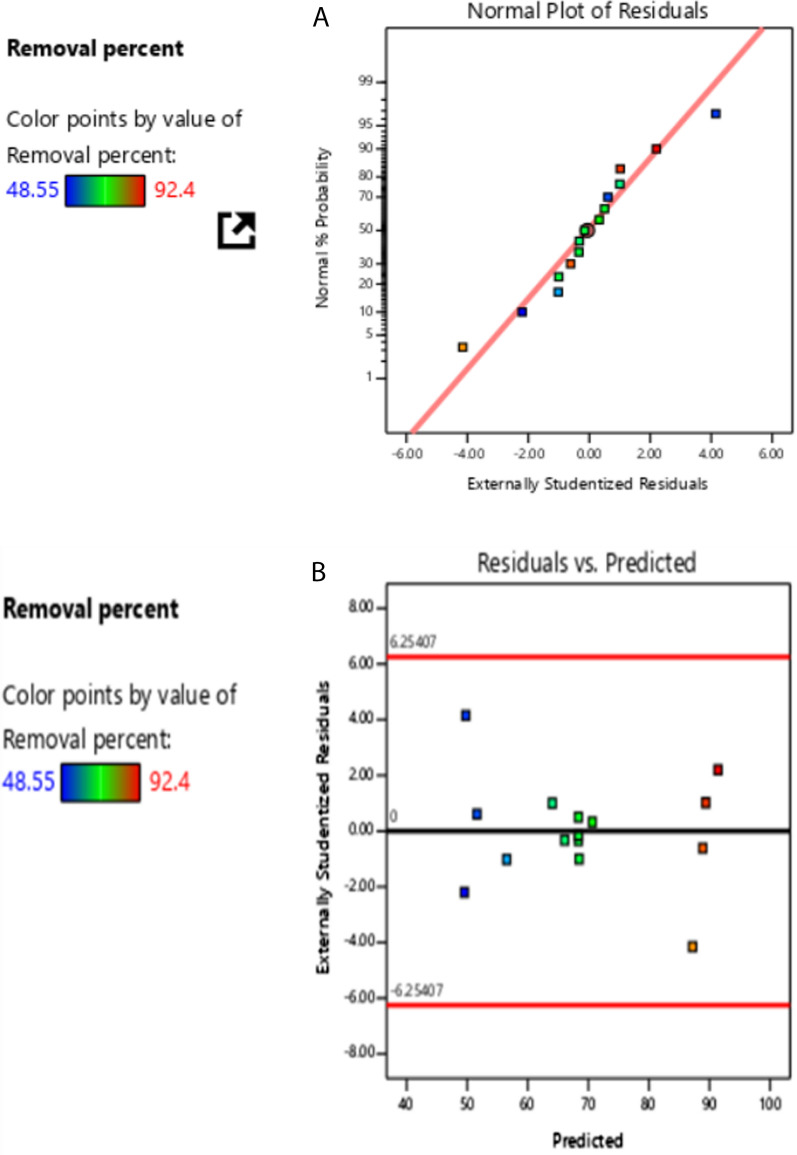
**a** Normal probability plot of residuals. **b** Plot of the residuals versus the predicted response

The ANOVA shows in Table 4 that the model is highly significant through the F value of 222.39 with a p-value less than 0.0001 which ensures the high significance. The lack of fit value is 6.769 which is not significantly proportionate to pure error 0.4 when p = 0.091, which is > 0.05 implies a good predictability. R^2^ is 0.997 close to Adjusted R^2^ which is 0.993 as the difference is less than 2 so this refers to a good predictability as well. Adequate Precision is 42.67 indicating an adequate signal. The adequacy of the model is observed to check if the model offers that the approximation to the true system was acceptable and none of the least squares regression assumptions are disobeyed. The normal probability plot of residuals checked the normality assumption and the normality assumption seemed to be acceptable as it provided a straight line as all the points were put on the diagonal. (Fig. 7a). Also, the plot of the residuals versus the predicted response showed a random scatter that seemed like a constant variance (Fig. 7b).

**Table 4:** ANOVA model for optimization

Source	Sum of Squares	df	Mean Square	F-value	p-value	
Model	2888.49	9	320.94	222.39	< 0.0001	Significant
A-pH	2792.66	1	2792.66	1935.12	< 0.0001	
B-Adsorbent conc	0.0105	1	0.0105	0.0073	0.9353	
C-drug conc	40.41	1	40.41	28.00	0.0032	
AB	3.08	1	3.08	2.13	0.2039	
AC	6.15	1	6.15	4.26	0.0939	
BC	4.45	1	4.45	3.08	0.1394	
A^2^	27.03	1	27.03	18.73	0.0075	
B^2^	10.60	1	10.60	7.35	0.0422	
C^2^	1.57	1	1.57	1.09	0.3442	
Residual	7.22	5	1.44			
Lack of Fit	6.77	3	2.26	10.10	0.0914	Not significant
Pure Error	0.4467	2	0.2233			
Cor. Total	2895.71	14

### Design parameters effect and their interactions

**Fig. 8:**
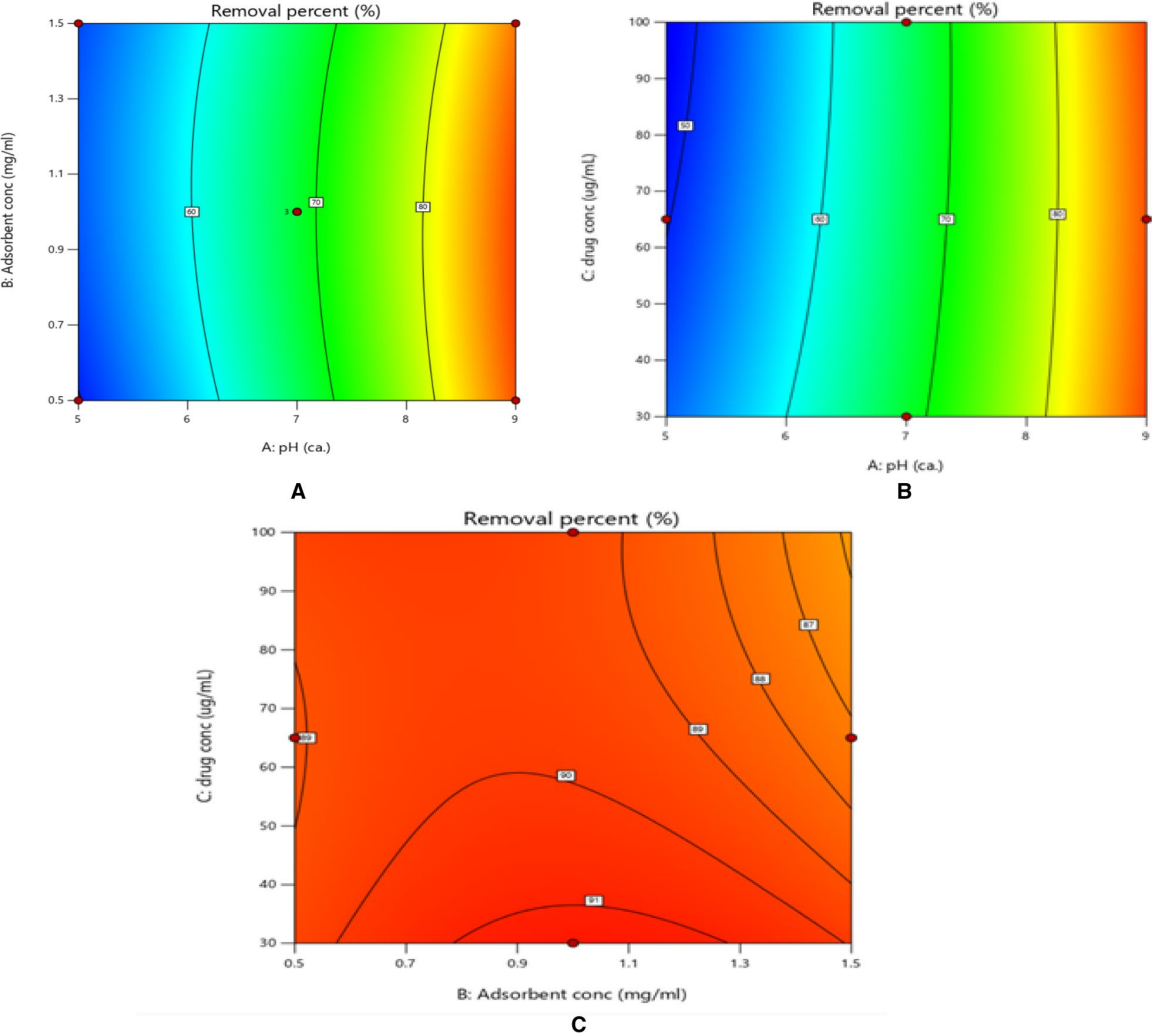
**a** Contour plot of the removal percentage% as a result of effect of adsorbent concentration versus pH. **b** Contour plot of the removal percentage% as a result of effect of drug concentration versus pH. **c** Contour plot of the removal percentage% as a result of effect of drug concentration versus adsorbent concentration

**Fig. 9:**
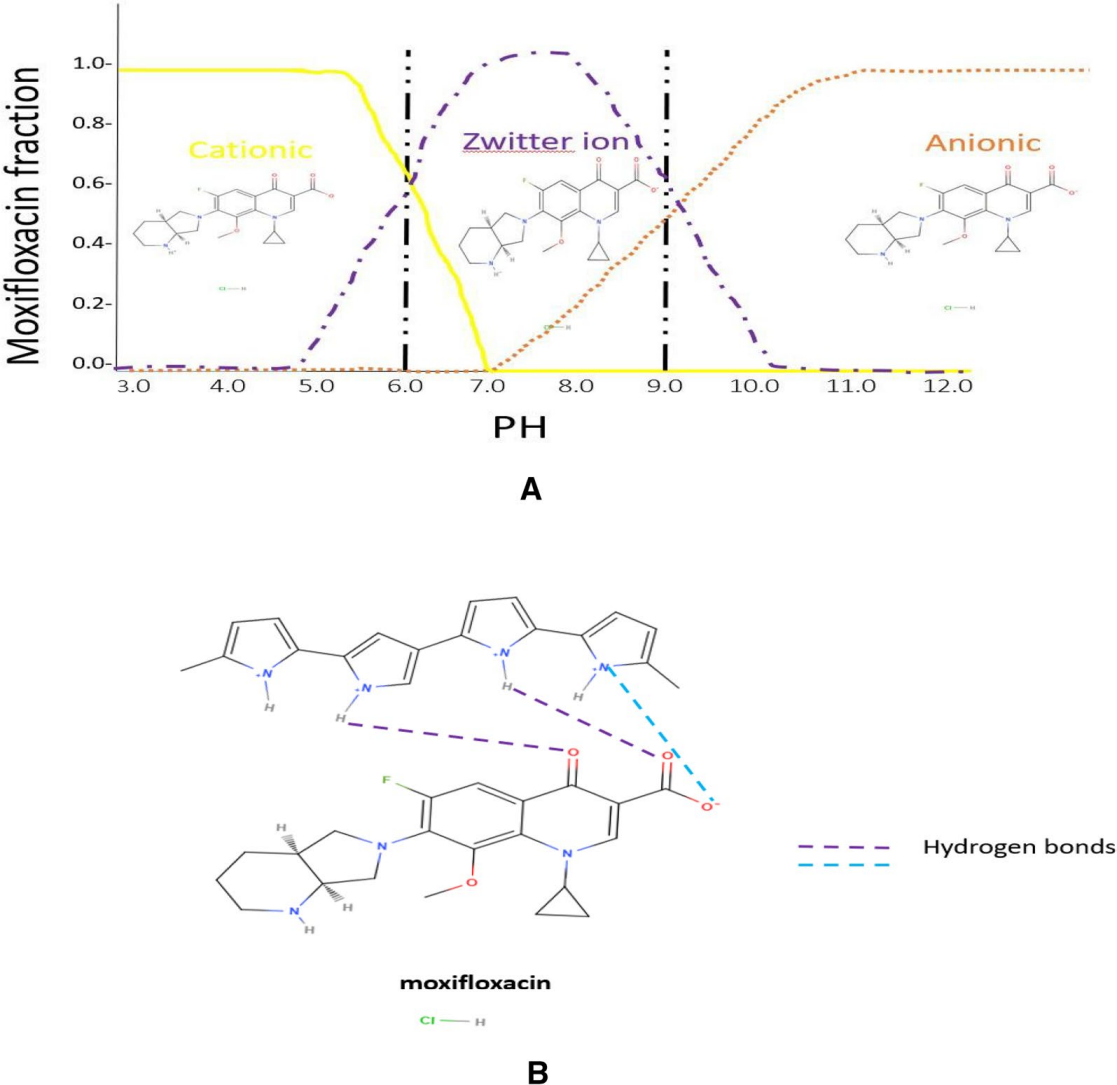
**a** pH effect on MXF adsorption. **b** Hydrogen bonds between adsorbent and adsorbate

Three-dimensional surface and contour plots are useful to visualize and learn the response and interactive effects of process variables [8]. Contour plots show the relationship between the 3 factors of pH, adsorbent concentration, and drug concentration, each 2 factors together to detect the effect of their interaction on the adsorption process. As shown in Fig. 8. An interaction between pH and adsorbent concentration, pH and drug concentration, and drug concentration and adsorbent concentration are shown as (a), (b), and (c) respectively. A slight decrease in drug concentration showed a slight increase in the response to the removal percentage, on the other hand, NP concentration (starting from 0.5 to 1.5 mg/mL) didn’t significantly affect the removal of MXF as enough binding sites were available even in low concentration of NP which shows a good advantage environmentally and low costs. A high removal percentage reaching up to 92.4% related to the previous conditions of the pH (factor a), nanoparticle adsorbent (factor b), and drug concentration (factor c). It was found that GRP-PPY-NP in alkaline media has the best adsorption power compared to acidic and neutral media. With a removal percentage of 92.4% of MFX from the water sample which can be explained as with high pH (alkaline 9 pH) the drug owns the negative charge of the COO- as it lost the H with the OH- group of alkaline media, then it becomes an anionic drug attracts positive charge (NH +) of GRP-PPY-NP with a strong hydrogen bond. (Fig. 9b) This explains the strong adsorption percentage and removal of the drug by GRP-PPY-NP. (Fig. 9), so the best conditions for MXF removal was 92.4% percent with 30 μg/mL of its concentration in pH 9 and adsorbent concentration of 1 mg/mL in 1 h.

### Adsorption isotherms

**Fig. 10:**
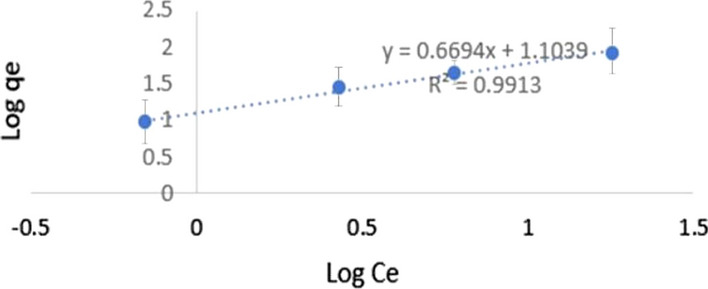
Freundlich adsorption isotherm using GRPPPY adsorbent

**Fig. 11:**
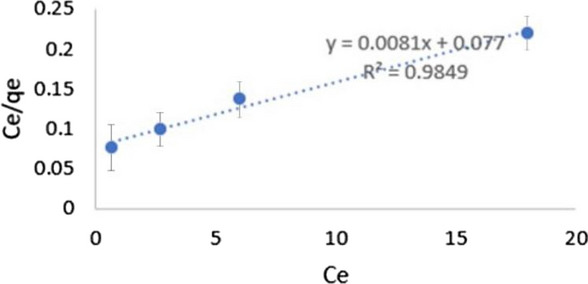
Langmuir adsorption isotherm using GRPPPY adsorbent

According to the isotherm parameters listed in Table 5, and from the linearity coefficients (R2 > 0.99), it was found that the Freundlich model only described the adsorption of MXF (Fig. 10) better than the Langmuir model (Fig. 11). As the value of 1 / n (0–1) can be used as an indication of the surface heterogeneity, and the closer the value to zero the higher the heterogeneity. Based on the parameters estimated using the Freundlich isotherm model (Table 5), it could be concluded that the adsorption was placed on a heterogeneous surface since the 1 / n value was in the range of 0–1.

**Table 5:** Langmuir and Freundlich isotherm equations and values

Model	Parameters	Values
Langmuir	qmax (mg/g)	123.4568
	Kl (L/mg)	0.000624
	RL	0.9938
	R^2^	0.9849
Freundlich	qmax (mg/g)	123.76
	R^2^	0.9913
	KF (mg/g (L mg)	12.7
	N	1.4938
	1/N	0.6694

### Adsorption effect on real water samples

When the optimized conditions were applied to clean real water from the Nile River of Zamalek- Egypt, the efficiency of adsorption in the real sample showed no significant change when compared to the results of the laboratory distilled water samples (both spiked with equal amounts of MXF, 30 μg/mL). The suggested adsorption approach was applied to real water samples containing MXF and because of the great efficiency of the GRP-PPY-NP process, the analysis that followed the adsorption process revealed results that were comparable to those of the clean genuine water samples.

### Reusability and sustainability

**Fig. 12:**
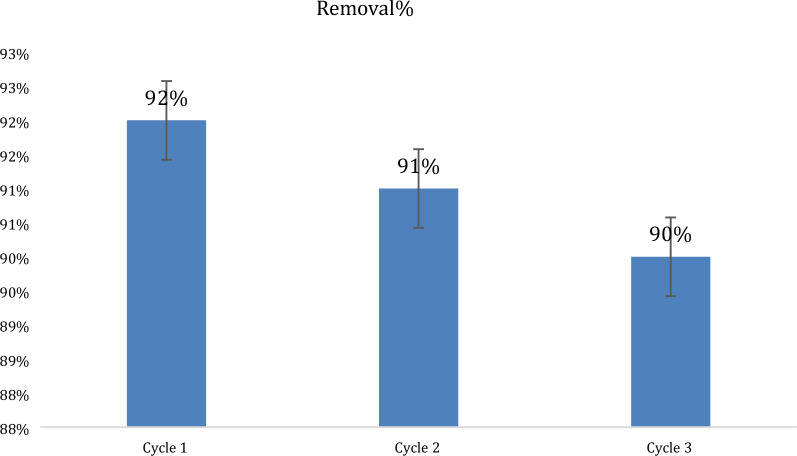
Bar chart for removal percentage power of NP after 3 cycles of washing

A washing process by methanol: acetonitrile 1:1 (v/v) equal ratios of 50 mL of solutions was applied for the NP for almost 12 h at room temperature in an orbital shaker at 200 rpm. Then rinsed three times with DW to remove any traces of desorbent agent and adsorbed MXF. Separated by filtration, the resulting desorbed GRP-PPY-NP was dried in air overnight and then reused for MXF. The recycling process aims to examine reusability and sustainability. GRP-PPY-NP are used for 3 cycles with no significant difference in adsorption and retained to almost 90% high efficacy in MXF removal which shows a great profitable advantage, high reusability and sustainability explained in a bar chart for removal percentage for each cycle (Fig. 12). In comparison with reported methods for NP reusability such as magnetic nanoparticles Fe_3_O_4_ with SiO_2_, the loss of adsorption capacity was about 20% for the removal of CIP and MXF [20] which the loss is greater than the current study, also in [24], about 88.72% of removal of MXF is achieved for the third cycle of CoFe_2_O_4_ NP which shows an advantage for GRP-PPY-NP reusability and recycling.

This study shows advantages over the reported methods due to the novelty of using GRP-PPY-NP for a pharmaceutical compound, it was used only for ions, heavy metals, or dyes. The study also shows advantages for MXF removal over other reported methods with other removal methods, which are explained in Table 6. It also shows the benefit of using graphene in combination with polypyrrole for a synergistic effect of adsorption which has been explained before. Furthermore, not using a high temperature to keep the environment safe in an eco-friendly manner shows a great benefit in comparison to other methods which use a high temperature of over 300 °C with a lower initial concentration of MXF. [3]. The other reported methods used a difficult method of preparation and long procedures [2]. A shorter time of contact with GRP-PPY-NP to save time and high efficiency also shows a benefit over the reported methods as they took more time for the process [33].

**Table 6:** The advantages of the current study over the reported studies

Removed matter	NP used	The drawback of the reported study over this current study	Citation
Mn^2+^	Graphene-polypyrrole nanoparticle	Used for metal ion removal, not for a pharmaceutical compound	[7]
Methylene Blue Dye and Bisphenol-A	Graphene-polypyrrole-magnetite nanocomposite	Used for dye and chemical compound removal, not for a pharmaceutical compound	[4]
Cu^2+^	MnO2-Coated Graphene/Polypyrrole Hybrids	Used for metal ion removal, not for a pharmaceutical compound	[45]
MXF	PectinFunctionalized Magnetic Nanoparticles	The maximum percentage of removal was attained as 89%	[6]
MXF	Molecularly imprinted polymers (MIPs)	High costs and difficult preparation	[17]
MXF	Biochars	Long preparation steps with high costs, time consumption, and high temperature/ non-eco-friendly methods were adopted	[3]

## Conclusion

In conclusion, this study showed a simple and effective method to remove MXF with 92.4% efficiency from wastewater using an adsorption technique by GRP-PPY-NP. A great efficacy and potential were obtained to remove this pharmaceutical product not only for dyes and ions as mentioned in previous reported methods which is considered the novelty of the study. With all the mentioned advantages and the ease of synthesis of the NP at room temperature to prepare an eco-friendly GRP-PPY-NP, it was suitable for environmental remediation and practical applications in wastewater treatment. Characterization studies were applied using FT-IR, SEM, XRD, BET, and HPLC under experimental conditions optimized through Box–Behnken design choosing three factors: pH, adsorbent concentration, and drug concentration in three levels while keeping the constant room temperature in 1 h time for green analysis. To sum up, the application of GRP-PPY-NP for MXF adsorption offers a promising road for addressing all the challenges related to pharmaceutical contamination in wastewater. Continued efforts in this research area can contribute to the development of efficient and sustainable solutions for water treatment and the challenge is to continue the investigations into how to use these studies in the pharmaceutical industry and water pipes to save the environment from continuous harm.

### Supplementary Information

Supplementary Material 1.

## Data Availability

All data generated or analyzed during this study are included in this published article.

## References

[1] Adeyemi JO, Ajiboye TO, Onwudiwe DC (2021). Mineralization of antibiotics in wastewater via photocatalysis. Water Air Soil Pollut.

[2] Ahmad M, Ahmadi SH, Jabbari V (2015). On-line microextraction of moxifloxacin using Fe3O4 nanoparticle-packed in-tube SPME. RSC Adv.

[3] Akhtar L, Ahmad M, Iqbal S, Abdelhafez AA, Mehran MT (2021). Biochars’ adsorption performance towards moxifloxacin and ofloxacin in aqueous solution: role of pyrolysis temperature and biomass type. Environ Technol Innov.

[4] Ama OM, Aigbe UO, Anku WW, Osibote OA, Pal K (2022). Degradation of methylene blue dye and Bisphenol-A using expanded Graphene-Polypyrrole-Magnetite nanocomposite. Top Catal.

[5] Anuma S, Mishra P, Bhat BR (2021). Polypyrrole functionalized Cobalt oxide Graphene (COPYGO) nanocomposite for the efficient removal of dyes and heavy metal pollutants from aqueous effluents. J Hazard Mater.

[6] Attallah OA, Al-Ghobashy MA, Nebsen M, Salem MY (2016). Adsorptive removal of fluoroquinolones from water by pectin-functionalized magnetic nanoparticles: process optimization using a spectrofluorimetric assay. ACS Sustain Chem Eng.

[7] Attia NF, Diab M, Attia AS, El-Shahat MF (2021). Greener approach for fabrication of antibacterial graphene-polypyrrole nanoparticle adsorbent for removal of Mn2+ from aqueous solution. Synth Met.

[8] Balasubramani K, Sivarajasekar N, Naushad M (2020). Effective adsorption of antidiabetic pharmaceutical (metformin) from aqueous medium using graphene oxide nanoparticles: equilibrium and statistical modelling. J Mol Liq.

[9] Barabadi H, Honary S, Ebrahimi P, Alizadeh A, Naghibi F, Saravanan M (2019). Optimization of myco-synthesized silver nanoparticles by response surface methodology employing Box-Behnken design. Inorg Nano Metal Chem.

[10] Barakan S, Aghazadeh V (2020). The advantages of clay mineral modification methods for enhancing adsorption efficiency in wastewater treatment: a review. Environ Sci Pollut Res.

[11] Blankenship LS, Mokaya R (2022). Modulating the porosity of carbons for improved adsorption of hydrogen, carbon dioxide, and methane: a review. Mater Adv.

[12] Butler S, Hollen SM, Cao L, Cui Y, Gupta J, Gutierrez H, Heinz TF, Hong SS, Huang J, Ismach A, Johnston-Halperin E, Kuno M, Plashnitsa VV, Robinson RD, Ruoff RS, Salahuddin S, Shan J, Shi L, Spencer MG, Goldberger JE (2013). Progress, challenges, and opportunities in two-dimensional materials beyond graphene. ACS Nano.

[13] Da Rosa Salles T, Da Silva Bruckamann F, Viana AR, Krause LMF, Mortari SR, Rhoden CRB (2022). Magnetic nanocrystalline cellulose: azithromycin adsorption and in vitro biological activity against melanoma cells. J Polym Environ.

[14] Da Silva Bruckmann F, Schnorr C, Da Rosa Salles T, Nunes FB, Baumann L, Müller EI, Silva L, Dotto GL, Rhoden CRB (2022). Highly efficient adsorption of tetracycline using chitosan-based magnetic adsorbent. Polymers.

[15] Elbalkiny HT, El-Zeiny MB, Saleh SS (2023). Analysis of commonly prescribed analgesics using. Environ Chem.

[16] Hamidi AA, Yilmaz Ş (2021). Antibiotic consumption in the hospital during COVID-19 pandemic, distribution of bacterial agents and antimicrobial resistance: a single-center study. J Surg Med.

[17] Hu G, Wu TT, Liu Z, Gao S, Hao J (2023). Application of molecular imprinting technology based on new nanomaterials in adsorption and detection of fluoroquinolones. Anal Methods.

[18] ICH Harmonized Tripartite Guidelines, Validation of Analytical Procedures: Text and Methodology Q2(R1), International Conference on Harmonization of Technical Requirments for Registeration of Pharmaceuticals for Human Use, Geneva, 2022.

[19] Ince M, Ince OK (2017). An overview of adsorption technique for heavy metal removal from water/wastewater: a critical review. Int J Pure Appl Sci.

[20] Jiang J, Jiang X, Zou Y, Zhai J, Ding W, Li H, Zheng H (2023). Facile synthesis of acid catalyzed sulfonic acid-amide functionalized magnetic sodium alginate and its efficient adsorption for ciprofloxacin and moxifloxacin. J Clean Prod.

[21] Kanakaraju D, Glass B, Oelgemöller M (2013). Titanium dioxide photocatalysis for pharmaceutical wastewater treatment. Environ Chem Lett.

[22] Kasisomayajula S, Qi X, Vetter C, Croes KJ, Pavlacky DA, Gelling VJ (2009). A structural and morphological comparative study between chemically synthesized and photopolymerized poly(pyrrole). J Coat Technol Res.

[23] Lee S, Lee S, Roh J (2021). Analysis of activation process of carbon Black based on structural parameters obtained by XRD analysis. Crystals.

[24] Liu H, Mi H, Meng Z, Sun F, Zhan R, Zhao H, He S, Zhou L (2021). Efficient moxifloxacin degradation by CoFe2O4 magnetic nanoparticles activated peroxymonosulfate: Kinetics, pathways and mechanisms. Chem Eng J.

[25] Malakootian M. Metronidazole adsorption on CoFe2O4/activated carbon@chitosan as a new magnetic biocomposite: modelling, analysis, and optimization by response surface methodology. 2021. www.academia.edu. https://www.academia.edu/54971938/Metronidazole_adsorption_on_CoFe2O4_activated_carbon_at_chitosan_as_a_new_magnetic_biocomposite_modelling_analysis_and_optimization_by_response_surface_methodology?hb-sb-sw=80155267

[26] Maleky S, Asadipour A, Nasiri A, Luque R, Faraji M (2021). Tetracycline Adsorption from Aqueous Media by Magnetically Separable Fe3O4@Methylcellulose/APTMS (Isotherm, Kinetic and Thermodynamic Studies). Res Square.

[27] Meimand MM, Jafari AJ, Nasiri A, Malakootian M (2020). Sulfur dioxide adsorption by Iron Oxide Nanoparticles@Clinoptilolite/HCl. J Air Pollut Health.

[28] Nasiri A, Malakootian M, Shiri MA, Yazdanpanah G, Nozari M. CoFe2O4@methylcellulose synthesized as a new magnetic nanocomposite to tetracycline adsorption: modeling, analysis, and optimization by response surface methodology. 2021. https://www.semanticscholar.org/paper/CoFe2O4%40methylcellulose-synthesized-as-a-new-to-and-Nasiri-Malakootian/5689905d7bb921d17fb648786efa53595fda8c51

[29] Nasiri A, Rajabi S, Hashemi M, Nasab H (2022). CuCoFe2O4@MC/AC as a new hybrid magnetic nanocomposite for metronidazole removal from wastewater: bioassay and toxicity of effluent. Separat Purif Technol.

[30] Rhoden CRB, Da Silva Bruckmann F, Da Rosa Salles T, Kaufmann CG, Mortari SR (2021). Study from the influence of magnetite onto removal of hydrochlorothiazide from aqueous solutions applying magnetic graphene oxide. J Water Process Eng.

[31] Riad S, Khattab FI, Salem H, Elbalkiny H. Ion-Selective membrane sensors for the determination of ciprofloxacin hydrochloride in water and. ResearchGate. 2014. https://www.researchgate.net/publication/287313113_Ion-Selective_membrane_sensors_for_the_determination_of_ciprofloxacin_hydrochloride_in_water_and_pharmaceutical_formulation

[32] Riad S, Salem H, Elbalkiny HT, Khattab FI (2015). Validated univariate and multivariate spectrophotometric methods for the determination of pharmaceuticals mixture in complex wastewater. Spectrochim Acta.

[33] Rubashvili I, Eprikashvili LG, Kordzakhia T, Zautashvili M, Pirtskhalava N, Dzagania MA (2019). Adsorptive removal study of the frequently used fluoroquinolone antibiotics - moxifloxacin and norfloxacin from wastewaters using natural zeolites. Mediterr J Chem.

[34] Singh RN, Sahoo S, Mishra U, Garnaik B, Sahoo SK, Hati D (2014). Stability indicating Rp-Hplc method development and validation of moxifloxacin. Int J Res Pharm Chem.

[35] Tan K, Hameed B (2017). Insight into the adsorption kinetics models for the removal of contaminants from aqueous solutions. J Taiwan Inst Chem Eng.

[36] The Unites States Pharmacopoeia and National Formulary. United States Pharmacoepeial Convention Inc., Rockville; 2021.

[37] Van Doorslaer X, Demeestere K, Heynderickx PM, Van Langenhove H, Dewulf J (2011). UV-A and UV-C induced photolytic and photocatalytic degradation of aqueous ciprofloxacin and moxifloxacin: reaction kinetics and role of adsorption. Appl Catalysis B Environ.

[38] Vishnu D, Dhandapani B (2020). Integration of Cynodon dactylonandMuraya koenigiiplant extracts in amino-functionalised silica-coated magnetic nanoparticle as an effective sorbent for the removal of chromium(VI) metal pollutants. IET Nanobiotechnol.

[39] Vishnu D, Dhandapani B. A review on the synergetic effect of plant extracts on nanomaterials for the removal of metals in industrial effluents. 2021. http://www.eurekaselect.com. https://www.eurekaselect.com/article/103588

[40] Vishnu D, Dhandapani B (2021). Synthesis of novel adsorbent by incorporation of plant extracts in amino-functionalized silica-coated magnetic nanomaterial for the removal of Zn2+and Cu2+from aqueous solution. J Environ Health Sci Eng.

[41] Vishnu D, Dhandapani B, Vaishnavi G, Preethi V (2022). Synthesis of tri-metallic surface engineered nanobiochar from cynodon dactylon residues in a single step - Batch and column studies for the removal of copper and lead ions. Chemosphere.

[42] Vishnu D, Rajendran A, Dhandapani B (2023). A potent insight into the microalgal and surface-modified magnetic microalgal biomass synthesis and treatment strategies in the removal of selenium and chromium metal ions. Energy Ecol Environ.

[43] Yang X, Wan Y, Zheng Y, He F, Yu Z, Huang J, Wang H, Ok YS, Jiang Y, Gao B (2019). Surface functional groups of carbon-based adsorbents and their roles in the removal of heavy metals from aqueous solutions: a critical review. Chem Eng J.

[44] Yehia AM, Elbalkiny HT, Riad S, El-Saharty YS (2020). Application of chemometrics for spectral resolving and determination of three analgesics in water samples. J AOAC Int.

[45] Zhang Y, Wang Y, Xue J, Tang C (2022). MnO2-Coated graphene/polypyrrole hybrids for enhanced capacitive deionization performance of Cu2+ removal. Ind Eng Chem Res.

[46] Zhao X, Gao X, Ding R, Huang H, Gao X, Liu B (2023). Post-synthesis introduction of dual functional groups in metal–organic framework for enhanced adsorption of moxifloxacin antibiotic. J Colloid Interface Sci.

