# Supplementary figures and images for: Optimization of graphene polypyrrole for enhanced adsorption of moxifloxacin antibiotic: an experimental design approach and isotherm investigation
Source: BMC Chem. 2024 Jun 13;18(1):113. doi: 10.1186/s13065-024-01208-0 (PMC11177460; doi:10.1186/s13065-024-01208-0)

**Supplementary material:**


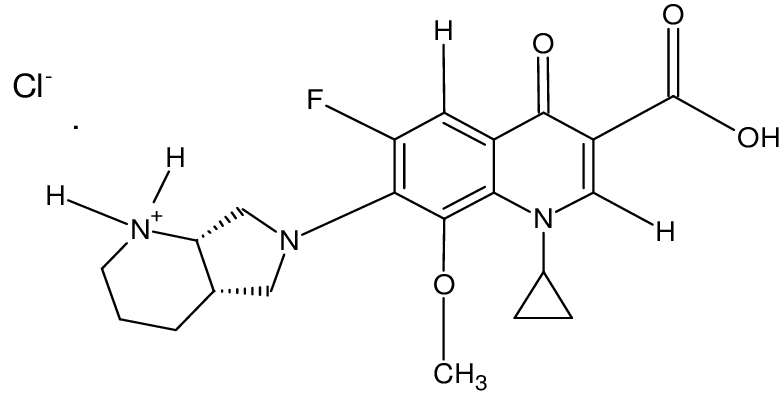


Supplementary material 1. Structure of Moxifloxacin HCl.

Supplement: Supplementary file 1 — Supplementary Material 1. [file 13065_2024_1208_MOESM1_ESM.docx]
